# Social Isolation, Healthy Habits, Inequality and Mental Health in the United States

**DOI:** 10.1007/s11482-023-10155-2

**Published:** 2023-03-10

**Authors:** Ignacio Amate-Fortes, Almudena Guarnido-Rueda, Diego Martínez-Navarro, Francisco J. Oliver-Márquez

**Affiliations:** Department of Economics and Business, University of Almeria, Carretera de Sacramento, s/n, 04120 Almeria, Spain

**Keywords:** Mental health, Suicide, Inequality, Social isolation, Healthy habits, I12, I14, I18, D63

## Abstract

The objective of this work is to deepen the analysis of the socioeconomic determinants of mental health, paying special attention to the impact of inequality, not only in income distribution but also in gender, racial, health and education inequality, social isolation, including new variables to measure loneliness, and healthy habits, on the mental health status. For this purpose, a cross-sectional model for a sample of 2735 counties in the United States is estimated using Ordinary Least Squares in its robust version to solve the detected heteroscedasticity problems. The results obtained show that inequality, social isolation and certain lifestyles, such as smoking or insomnia, are detrimental to mental health, while sexual activity prevents mental distress. On the other hand, poor counties suffer more cases of suicide, with food insecurity being the main problem for mental health. Finally, we found detrimental effects of pollution on mental health.

## Introduction

In recent decades, the role of mental health has grown in importance, not only for the scientific community, but also for policy makers as reflected by the fact that it has been incorporated into the Sustainable Development Goals. It is important to note that depression is one of the leading causes of disability and that suicide is the leading cause of death in the population between 15 and 29 years of age. In fact, according to the World Health Organization (WHO), more than 700,000 people die by suicide each year.

The United States is not immune to this problem and with a mortality rate of 16.1 per 100,000 inhabitants, it is one of the countries with the highest suicide rates. For all these reasons, in this paper we propose to analyze the socioeconomic determinants of mental health in the United States. To this end, we base our analysis on three pillars: firstly, inequality, understood in a broad sense, i.e., inequality in income distribution, gender, race, health, education and the labor market. This in-depth analysis of the incidence of inequality on mental health is the main novelty of this work. Second, we use several variables as proxies for social isolation to test how they affect mental health. In this sense, the use of new variables such as teleworking or driving alone every day to work is another important novelty of this article. Finally, the main lifestyle habits are analyzed to contribute to the analysis of the effect of these variables on mental health.

For this purpose, a robust cross-sectional model was estimated for a sample ranging from 1790 to 2735 U.S. counties (depending on the availability of data for certain variables). The results obtained show that inequality in all its aspects is indeed a risk factor for mental disorders, although social isolation is perhaps more important as an explanatory variable. Finally, tobacco addiction and insomnia are shown to be the habits most detrimental to mental health.

The second empirical analysis establishes the theoretical framework and then, in the third section, explains the model and discusses the results. Finally, in the fourth section, the conclusions are developed.

## Theoretical Framework

The economic literature has extensively studied the effects of inequality on different health outcomes (Pickett & Wilkinson, 2015; Matthew & Brodersen, 2018), and among these, some authors have addressed the relationship between inequality and mental health. Thus, works such as Burns et al. (2017), have analyzed the relationship between inequality in income distribution and certain mental disorders. However, as pointed out by Patel et al. (2018), a review of the papers published on the relationship between income inequality and mental health shows inconsistent results, with only one third of them concluding that inequality in income distribution is a risk factor for mental health.

Less studied is the case of the association between other forms of inequality and the prevalence of mental disorders. In this sense, there is a lack of work addressing the incidence of gender inequality on mental health (Yu, 2018). Even so, we can highlight the works of Hopcroft and Bradley (2007), and Van de Velde et al. (2013), who perform a macro-level analysis of the effects of gender inequality on mental health. As with inequality in income distribution, research on the effects of gender inequality on mental health reflects inconclusive results (Hopcroft & Bradley, 2007; Seedat et al., 2009; Van de Velde et al., 2013; Hagen & Rosenstrôm, 2016).

Most papers that have studied racial inequality as a risk factor for mental health have measured this racial inequality through discrimination (Brown et al., 2000; Lewis et al., 2015; Wallace et al., 2016; Mouzon et al., 2017; Williams, 2018). In this case, the results are indeed conclusive and point out that racial discrimination negatively affects mental well-being. Our work aims to delve deeper into the impact of racial inequality on mental health, measuring this inequality through the unequal distribution of poverty across races.

Regarding social isolation as a determinant of mental health, there is a broad consensus from researchers about the positive impact of interpersonal relationships on mental well-being (Almedom, 2005; Bassett & Moore, 2013), However, an associated problem encountered by researchers is that it is unclear how social isolation, loneliness, and other related concepts should be measured when analyzing their effect on mental health (Windle et al., 2011; Courtin & Knapp, 2015; Rhode et al., 2016; Chirstiansen et al., 2021). Therefore, we propose new measures of social isolation such as teleworking and driving alone to work. In doing so, we aim to give robustness to the results obtained by the already published works.

Finally, the economic literature has also paid attention to the association between healthy habits and mental health. Thus, authors such as Reid et al. (2009), Taylor et al. (2011), Milojevich and Lukowaki (2016), Chattu et al. (2018), Sullivan and Ordiah (2018), and Merikanto and Partonen (2021) among others warn of the adverse effects of insomnia on mental health. Also, the impact of tobacco and alcohol addiction on mental health has aroused the interest of researchers, highlighting the works on the adolescent population by Mason et al. (2008), Balogun et al. (2014), Skogen et al. (2014), and Ferreira et al. (2019). Likewise, the relationship between obesity, physical activity, and mental health has been analyzed (Kivimâki et al., 2009). However, the mechanisms linking obesity and mental illness are unclear (Avila et al., 2015). Thus, there are authors who point out that mental disorders are the cause of obesity (Nicholson, 1946), others speak of a bidirectional relationship (Cameron et al., 2012) and others point to obesity as a risk factor for mental health (De Hert et al., 2011).

Therefore, as mental disorders cannot be explained solely through genetic factors (Sanders et al., 1999; Sullivan et al., 2000; Fava & Kendler, 2000), and given the importance of socioeconomic determinants, we propose, from here on, to continue to deepen the analysis of the incidence of these factors on mental health, with emphasis on inequality, social isolation and healthy living habits.

## Empirical Analysis

A cross-sectional linear model has been estimated to analyze whether social isolation, lifestyle, and inequality, broadly understood, observed in each North American county have any effect on mental health in the United States. The mental health data were obtained from the County Health Rankings & Roadmaps, University of Wisconsin Health Institute, and refer to 2019. In this sense, we have worked with a database of 3,218 U.S. counties, which is almost 100% of all counties in the U.S. Even so, the inequality and mental health data by counties have only allowed us to use a sample between 1,790 and 2,735 counties, depending on the inequality and mental health measure used. In any case, the sample used is representative of the overall U.S. situation.

**Fig. 1:**
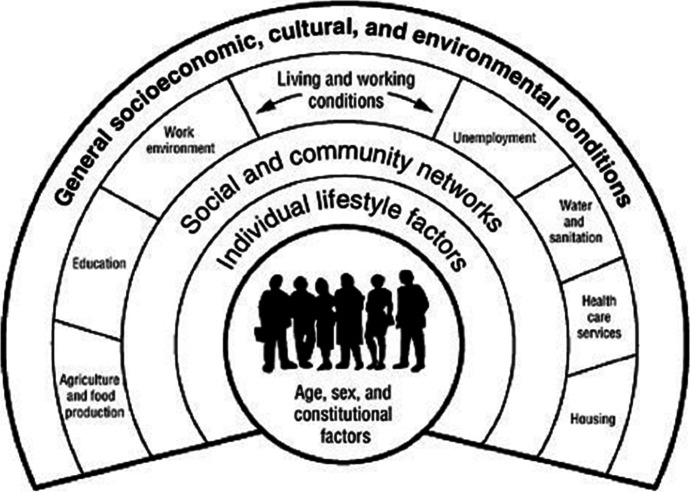
The Dalghren-Whitehead model of determinants in health. Source: Dalghren and Whitehead (1991)

This study adapts the classic model of Dalghren and Whitehead (1991) for a comparative analysis between counties in the United States. The model of these two economists has been widely used and shows the determinants of health in concentric layers, from structural determinants (external layer) to individual lifestyles (internal layer), placing at the center the characteristics of individuals that cannot be modified, such as sex, age or constitutional factors (Fig. 1).

According to these authors, individuals are endowed with risk factors such as age, sex and other genetic factors that influence their potential for ultimate health. Likewise, personal behaviors and lifestyles also play a role. People who are economically disadvantaged tend to exhibit behaviors that depart from healthy living, such as smoking, alcohol and drug abuse, and poor diet. On the other hand, labor and environmental conditions, and access to basic services constitute another set of determinants of health status. Differences in housing conditions, occupational risks, whether one has a job, and the possibility of having free, quality education, basic health services, and infrastructure access to drinking water, sewage systems, paved roads, are key factors in the differences in health shown by different social groups. Finally, the economic, cultural and environmental conditions prevailing in society as a whole, as well as the economic situation of the country, will also affect the health outcomes of the population as a whole.

In our case, we adapt this model to analyze the socioeconomic determinants of mental health.

- A. Data

The variables used in this work are summarized in the following table (Table 1):

**Table 1:** Variable definitions and summary statistics

Variable	Description	Obs.	Mean	Std. Dev.	Min	Max
Poor mental health days	It measures the average number of mentally unhealthy days reported in past 30 days during 2019. Sources: US State and Local Health Agencies. Source: County Health Rankings & Roadmaps 2022 (University of Wisconsin Population Health Institute). https://www.countyhealthrankings.org Accessed on July 28, 2022.	3143	4.89	0.69	3.24	7.46
Frequent mental distress	Percentage of adults reporting 14 or more days of poor mental health per month (age-adjusted). Frequent mental distress is a derived measure of poor mental health days. It provides a slightly different picture that emphasizes those experiencing more chronic and likely severe mental health problems. Source: County Health Rankings & Roadmaps 2022 (University of Wisconsin Population Health Institute). https://www.countyhealthrankings.org Accessed on August 19, 2022.	3141	0.16	0.03	0.097	0.26
Suicide	Number of deaths by suicide per 100,000 population (age-adjusted). Suicide serves as an important measure of the mental health of a county’s population. Outside of the impact on the emotional and mental health of surviving friends, family members, and loved ones, suicide also has an economic impact and costs the United States an estimated $70 billion per year (Centers for Disease Control and Prevention. Suicide Prevention – Facts About Suicide. Last August 30, 2021. Accessed on August 22, 2022. https://www.cdc.gov/suicide/facts/index.htm Source: County Health Rankings & Roadmaps 2022 (University of Wisconsin Population Health Institute). https://www.countyhealthrankings.org Accessed on August 19, 2022.	2433	18.94	7.88	4.98	155.37
Mean relative income	It is defined as the county inflation-adjusted mean household income relative to State mean household income. The data refers to 2020. It is a measure of inequality that aims to study whether poorer populations within a State are more vulnerable to the effects of the pandemic. Source: Prepared by the authors based on data from the United States Census Bureau. https://www.census.gov Accessed on July 21, 2022.	3081	1.16	0.25	0.58	2.74
Inequality	Inequality index by county. The following 5 inequality indexes have been used: • Gini Index (Income inequality). Measure of inequality in the distribution of the county´s households’ income. The value of the index varies between 0 and 1. Source: American Community Survey 2020 (5-Year Estimates). United States Census Bureau. https://www.census.gov Accessed on July 18, 2022.	3221	0.45	0.04	0.08	0.70
	• 80/20 Index (Income inequality). A measure of inequality that relates the percentage of the county’s households´ income obtained by the top 20% of income to the bottom 20%. Source: Own elaboration based on American Community Survey 2020 (5-Year Estimates). United States Census Bureau. https://www.census.gov Accessed on July 18, 2022.	3210	14.10	6.62	4.72	190.22
	• Gender Pay Gap (Gender inequality). It is expressed as “cents on the dollar,“ or women’s median earnings in cents compared to every dollar (100 cents) of men’s median earnings. Women’s median earnings are the level of earnings where half of full-time, year-round, female workers are earning more than this value, and half are earning less. Men’s median earnings follow the same definition for male workers. The wage gap still exists despite the fact that women make up the majority of college-educated adults in the U.S. and that decades after the Equal Pay Act of 1963 outlawed paying men and women different wages for similar work. Source: County Health Rankings & Roadmaps 2022 (University of Wisconsin Population Health Institute). https://www.countyhealthrankings.org Accessed on July 28, 2022.	3137	0.77	0.098	0.42	1.57
	• Female poverty (Gender inequality). It measures the percentage of women below the poverty thresholds out of the total number of poor people. As noted by Kawachi et al. (1999), and Milner et al. (2021), gender inequality is associated with worse mortality outcomes, poorer self-rated health, and greater disability. Source: Own elaboration based on American Community Survey 2020 (5-Year Estimates). United States Census Bureau. https://www.census.gov Accessed on July 19, 2022.	3220	0.56	0.05	0.20	0.81
	• Black poverty (racial inequality). It is the percentage of black population below the poverty threshold out of the total poor population. Source: Own elaboration based on American Community Survey 2020 (5-Year Estimates). United States Census Bureau. https://www.census.gov Accessed on July 19, 2022.	2753	0.15	0.21	0	0.98
	• Racial segregation (racial inequality). It refers to the degree to which black and white residents live separately from one another in a county. The residential segregation index ranges from 0 (complete integration) to 100 (complete segregation). The elimination of discriminatory policies and practices has had an impact on acts of racism, but has had little effect on structural racism, such as residential segregation, resulting in persistent structural inequalities. Residential segregation is a key determinant of racial differences in socioeconomic mobility and, in addition, can create social and physical risks in residential settings that negatively affect health. Although this research field is gaining interest, structural forms of racism and its relationship to health inequalities remain understudied (Gee & Ford, 2011). These authors and Kramer and Hogue (2009) assert that residential segregation is considered a root cause of health disparities in the U.S. and has been linked to poor health outcomes, such as mortality, a wide variety of reproductive, infectious, and chronic diseases, and other adverse conditions. Source: County Health Rankings & Roadmaps 2022 (University of Wisconsin Population Health Institute). https://www.countyhealthrankings.org Accessed on July 28, 2022.	2078	49.1	16.62	0.51	97.01
Mental health providers	Refers to the number of mental health providers per 1,000 population. Access to care requires not only financial coverage, but also access to providers. Source: County Health Rankings & Roadmaps 2022 (University of Wisconsin Population Health Institute). https://www.countyhealthrankings.org Accessed on July 28, 2022.	2946	3.04	17.29	0	478.57
Uninsured	It is the percentage of the population under age 65 without health insurance coverage. A person is uninsured if they are currently not covered by insurance through a current/former employer or union, purchased from an insurance company, Medicare, Medicaid, Medical Assistance, any kind of government-assistance plan for those with low incomes or disability, TRICARE or other military health care, Indian Health Services, VA, or any other health insurance or health coverage plan. More than 8% of the population remains uninsured in the U.S. Lack of health insurance coverage is a major barrier to accessing needed health care and maintaining economic security and using it is intended to test whether it has had any effect on the mental health status. Source: County Health Rankings & Roadmaps 2022 (University of Wisconsin Population Health Intistute). https://www.countyhealthrankings.org Accessed on July 28, 2022	3221	0.12	0.05	0	0.36
University	It measures the percentage of the population with university studies. The use of this variable is intended to test whether more education influences mental health. As mentioned above, the aim is to test whether education improves health equity, as argued by Amate-Fortes et al. (2020). The data refer to 2020. Source: United States Census Bureau. https://www.census.gov Accessed on July 21, 2022.	3221	0.16	0.07	0	0.57
Unemployment	Unemployment rate in 2020 by county. The goal is to verify if higher unemployment rate has any effect on mental health. Source: United States Census Bureau. https://www.census.gov Accessed on July 19, 2022.	3221	0.05	0.03	0	0.35
Bad health	It measures the percentage of adults in a county who consider themselves to be in poor or fair health (age adjusted) during 2019. Source: County Health Rankings & Roadmaps 2022 (University of Wisconsin Population Health Institute). https://www.countyhealthrankings.org Accessed on July 28, 2022.	3221	0.20	0.06	0	0.45
Sleep	Percentage of adults who report fewer than 7 h of sleep on average per day(age-adjusted) during 2018. Sleep is an important part of a healthy lifestyle, and a lack of sleep can cause psychiatric disorders such as depression and anxiety, risky behavior, and even suicide. Source: County Health Rankings & Roadmaps 2022 (University of Wisconsin Population Health Institute). https://www.countyhealthrankings.org Accessed on July 28, 2022.	3143	0.37	0.04	0	0.49
Smoke	Adult Smoking is the percentage of the adult population in a county who both report that they currently smoke every day or some days and have smoked at least 100 cigarettes in their lifetime. Each year, according to US Department of Health and Human Services, approximately 480,000 premature deaths can be attributed to smoking. Source: County Health Rankings & Roadmaps 2022 (University of Wisconsin Population Health Institute). https://www.countyhealthrankings.org Accessed on July 28, 2022.	3221	0.20	0.05	0	0.43
Obesity	It is based on responses to the Behavioral Risk Factor Surveillance Survey (BRFSS) and is the percentage of the adult population (ages 18 and older) that reports a body mass index (BMI) greater than or equal to 30 kg/m^2^. Participants are asked to self-report their height and weight. From these reported values, BMIs for the participants are calculated. Source: County Health Rankings & Roadmaps 2022 (University of Wisconsin Population Health Institute). https://www.countyhealthrankings.org Accessed on July 28, 2022.	3221	0.35	0.07	0	0.51
Inactivity	Physical Inactivity is based on responses to the Behavioral Risk Factor Surveillance Survey and is the percentage of adults ages 18 and over reporting no leisure-time physical activity in the past month. Physical inactivity is not only associated with individual behavior, but also with community conditions, such as spending on recreational activities, access to infrastructure, and poverty (Lee et al., 2017). Source: County Health Rankings & Roadmaps 2022 (University of Wisconsin Population Health Institute). https://www.countyhealthrankings.org Accessed on July 28, 2022.	3221	0.30	0.07	0	0.52
Alcohol	Excessive Drinking measures the percentage of a county’s adult population that reports binge or heavy drinking in the past 30 days. The Centers for Disease Control and Prevention (2009) warn of significant adverse health effects. Source: County Health Rankings & Roadmaps 2022 (University of Wisconsin Population Health Institute). https://www.countyhealthrankings.org Accessed on July 28, 2022.	3221	0.19	0.04	0	0.30
Sexual transmitted infections (STI)	It the number of newly diagnosed chlamydia cases per 100,000 population in a county. We use this indicator as a proxy variable for the population’s sexual activity. This will allow us to analyze whether those counties with a greater sexual activity also have a better mental health. Source: County Health Rankings & Roadmaps 2022 (University of Wisconsin Population Health Institute). https://www.countyhealthrankings.org Accessed on July 28, 2022.	3025	418.3	293.6	0	3848.9
Density	Variable that measures the population per square mile of land area by county. The aim is to verify whether the larger counties, which tend to have a higher population density, have a better mental health. Source: United States Census Bureau. https://www.census.gov Accessed on July 22, 2022.	3182	258.4	1706.3	0	69468.4
Associations	Number of membership associations per 10,000 population in 2019. Source: County Health Rankings & Roadmaps 2022 (University of Wisconsin Population Health Intistute). https://www.countyhealthrankings.org Accessed on August 19, 2022.	3143	11.44	5.91	0	55.4
Work from home (WFH)	Percentage of workers who teleworked in 2018. The objective of using this variable is to test whether social isolation has an impact on mental health. Source: National Association of Realtors. https://www.nar.realtor/research-and-statistics/research-reports/work-from-home-counties Accessed on August 3, 2022.	3092	4.75	2.83	0	27.3
Driving	Percentage of the workforce that drives alone to work. Source: County Health Rankings & Roadmaps 2022 (University of Wisconsin Population Health Institute). https://www.countyhealthrankings.org Accessed on August 19, 2022.	3143	0.79	0.08	0	0.99
Broadband	Percentage of households with broadband internet connection. Source: County Health Rankings & Roadmaps 2022 (University of Wisconsin Population Health Instute). https://www.countyhealthrankings.org Accessed on August 19, 2022.	3141	0.78	0.08	0.33	0.97
Severe housing problems	Percentage of households with at least 1 of 4 housing problems: overcrowding, high housing costs, lack of kitchen facilities, or lack of plumbing facilities. Source: County Health Rankings & Roadmaps 2022 (University of Wisconsin Population Health Institute). https://www.countyhealthrankings.org Accessed on August 19, 2022.	3143	0.13	0.04	0	0.70
Food	Percentage of the population lacking adequate access to food in 2019. Source: County Health Rankings & Roadmaps 2022 (University of Wisconsin Population Health Institute). https://www.countyhealthrankings.org Accessed on August 19, 2022.	3141	0.13	0.04	0.03	0.29
Pollution	Air Pollution - Particulate Matter is a measure of the fine particulate matter in the air. It is reported as the average daily density of fine particulate matter in micrograms per cubic meter. Fine particulate matter is defined as particles of air pollutants with an aerodynamic diameter less than 2.5 micrometers (PM2.5). Fuente: County Health Rankings & Roadmaps 2022 (University of Wisconsin Population Health Institute). https://www.countyhealthrankings.org Accessed on July 28, 2022.	3115	8.02	1.65	2.5	20.9

Source: Own elaboration

- B. The model

A linear model was developed and estimated through Ordinary Least Squares in its robust version of variances and covariances, since when the Breusch-Pagan test was performed, the p-value obtained showed the presence of heteroscedasticity. The model was estimated without a constant term. Although the decision to use a constant term or not is a problem that generates much discussion (Casella, 1983), nevertheless, there are circumstances in which it is appropriate or even necessary not to use the error term. As Eisenhauer (2003) points out, in the case where the dependent variable is zero if the vector of independent variables is also zero, the error term can be omitted. This is the case of the estimated model where variables such as population density are used. If this variable had a value equal to zero, the variables measuring mental health status would also have a value equal to zero.

The model used is as follows:

1 $$\begin{array}{lllll}MENTALHEALTH={\beta }_{1}INCOME+{\beta }_{2}INEQUALITY+{\beta }_{3}MHP+{\beta }_{4}UNINSURED+ \\ {\beta }_{5}UNIVERSITY+{\beta }_{6}UNEMPLOYMENT+{\beta }_{7}BADHEALTH+{\beta }_{8}SLEEP+ \\ {\beta }_{9}SMOKE+{\beta }_{10}OBESITY+{\beta }_{11}INACTIVITY+{\beta }_{12}ALCOHOL{+\beta }_{13}STI+ \\ {\beta }_{14}DENSITY+{\beta }_{15}ASSOCIATIONS+{\beta }_{16}WFH+{\beta }_{17}DRIVING+ \\ {\beta }_{18}BROADBAND+{\beta }_{19}SHP+{\beta }_{20}FOOD+{\beta }_{21}POLLUTION+{\mu }_{I}\end{array}$$

Where,

*Mentalhealth* is the dependent variable. In this sense, three variables that reflect the mental health status have been used, each of them implying an aggravation of mental disorders. Thus, first, the variable “Poor mental health days” has been used, which measures the average number of days of mental unhealthiness reported in the last 30 days during 2019. The second variable used is “Frequent mental distress” which reflects the percentage of adults reporting 14 or more poor mental health days per month (age-adjusted), therefore, it emphasizes the population experiencing more chronic and probably more severe mental health problems. Finally, the variable “Suicide” was used, which measures the number of suicide deaths per 100,000 population (age-adjusted). This variable reflects the extreme case of a mental health problem. The objective, therefore, is to analyze how the independent variables used affect mental health and how these effects change as mental illness worsens.

*Income* measures the average real income per household in the county in relation to the average real income per household in the state. It is, therefore, a first variable that measures inequality, in this case, between counties.

*Inequality* is one of the explanatory variables on which we have focused the objective of this work, i.e., the aim is to analyze how inequality within each county affects mental health. In this sense, we have tried to analyze inequality in a broad sense, that is, not only inequality in income distribution, but also gender inequality and racial inequality. For this purpose, six measures of inequality were used:

- The Gini index and the 80/20 ratio have been used to measure inequality in income distribution.
- In terms of gender inequality, the Gender Pay Gap, which measures the average earnings of women in relation to men, and the female poverty variable, which refers to the percentage of poor women in relation to the total poor population, have been used.
- Racial inequality is measured by the percentage of the African American poor population out of the total population below the poverty line, and by racial segregation, i.e., the degree to which black and white residents live separately from each other in a county.

*MHP* refers to the number of mental health providers per 1,000 population. It is therefore a proxy variable for access to mental health care, since access to care requires not only financial coverage, but also access to providers.

*Uninsured* refers to the percentage of people under age 65 who did not have health insurance in 2019. This is a proxy variable for health inequality in terms of health coverage. Therefore, its use is intended to strengthen the analysis of the effects of inequality on mental health.

*University* measures the percentage of the population with university studies. As with the previous variable, this is a proxy variable for educational inequality and will allow us to delve deeper into inequality as a determinant of mental health.

*Unemployment* measures the unemployment rate in 2020. This variable shows, on the one hand, the inequality in the labor market between counties and, on the other hand, the lack of income.

*Badhealth* measures the percentage of adults in a county who consider themselves to be in poor or fair (age-adjusted) health during 2019. The purpose of using this variable is to test whether physical health has a relationship to mental health.

*Sleep* refers to the percentage of adults reporting having slept less than 7 h on average per day (age-adjusted) during 2018. Sleep is an important part of a healthy lifestyle, and by employing this variable we try to analyze whether lack of sleep can cause psychiatric disorders.

*Smoke* refers to the percentage of a county’s adult population reporting smoking every day or some days, and having smoked at least 100 cigarettes in their lifetime, in 2019. We use “Smoke” as a proxy variable for addiction.

*Obesity* is the percentage of the adult population with a body mass index (BMI) equal to or greater than 30 kg/m^2^. The objective is to see if obesity is a cause of poor mental health.

*Inactivity* is the percentage of adults aged 18 years and older reporting no leisure-time physical activity in the last month during 2019.

*Alcohol* measures the percentage of a county’s adult population reporting binge drinking in the past 30 days, during 2019.

*STI* refers to sexually transmitted diseases measured through the number of newly diagnosed chlamydia cases per 100,000 population in 2019. We use “STI” as a proxy variable for sexual activity.

*Density* is the population density of the county in 2020, i.e., number of inhabitants per km^2^. This is the first variable that will allow us to analyze the effects of social isolation on mental health.

*Associations* measures the number of membership associations per 10,000 inhabitants in 2019.

*WFH* refers to “work from home,“ i.e., the percentage of people who teleworked in 2018. Teleworking prevents physical contact with coworkers and is therefore a good proxy for loneliness.

*Driving* measures the percentage of the labor force that drives alone to work.

*Broadband* is the percentage of households with a broadband Internet connection. We use this variable as a proxy for home Internet use.

SHP is “Severe housing problems” and refers to the percentage of households with at least 1 of these 4 housing problems: overcrowding, high housing costs, lack of kitchen facilities or lack of plumbing facilities. With this, we want to check whether these types of problems lead to poorer mental health.

*Food* measures the percentage of the population lacking adequate access to food in 2019.

*Pollution* refers to air pollution - particulate matter and is a measure of fine particles in the air. It is presented as the daily average density of fine particles in micrograms per cubic meter. Fine particulate matter is defined as air pollutant particles with an aerodynamic diameter of less than 2.5 micrometers (PM2.5).

- C. Results and Discussion

As mentioned above, the model was estimated by OLS in its robust version to solve the problem of heteroscedasticity detected. Eighteen estimates have been made, resulting from the use of 6 different measures of inequality and the three dependent variables used to characterize mental health. The results are reflected in the following tables (Tables 2, 3, and 4):

**Table 2:** Results of the estimations (income inequality)

	Poor mental health days		Frequent mental distress		Suicide	
	Gini	80/20	Gini	80/20	Gini	80/20
Mean relative income	0.19^***^ (5.41)	0.20^***^ (5.79)	0.003^***^ (2.77)	0.003^***^ (3.17)	-2.45^***^ (-2.94)	-2.63^***^ (-3.29)
Inequality	0.79^***^ (4.32)	0.001 (0.58)	0.02^***^ (4.09)	0.00004 (0.57)	-1.00 (-0.20)	0.10 (0.56)
Mental health provider	-0.0003^*^ (-1.66)	-0.0003 (-1.47)	-0.00007 (-0.97)	-0.00005 (-0.80)	-0.006 (-0.51)	-0.006 (-0.49)
Uninsured	-1.001^***^ (-5.90)	-0.99^***^ (-5.72)	-0.03^***^ (-6.33)	-0.03^***^ (-6.18)	23.82^***^ (5.16)	24.11^***^ (4.94)
University	-0.31^**^ (-1.99)	-0.17 (-1.10)	-0.007 (-1.40)	-0.003 (-0.59)	-10.76^**^ (-2.36)	-11.92^**^ (-2.31)
Unemployment	0.08 (0.21)	0.17 (0.45)	-0.009 (-0.71)	-0.006 (-0.48)	36.66^*^ (1.65)	35.88^**^ (1.94)
Bad health	3.91^***^ (10.37)	4.09^***^ (10.69)	0.14^***^ (11.63)	0.15^***^ (11.99)	-13.49 (-1.18)	-14.72 (-1.23)
Sleep	5.19^***^ (19.05)	5.30^***^ (19.19)	0.14^***^ (16.15)	0.14^***^ (16.25)	19.41^*^ (1.80)	20.50^*^ (1.81)
Smoke	8.38^***^ (24.74)	8.34^***^ (24.22)	0.33^***^ (29.78)	0.33^***^ (29.19)	51.12^***^ (3.01)	51.52^***^ (3.05)
Obesity	-1.52^***^ (-6.23)	-1.53^***^ (-6.18)	-0.03^***^ (-4.20)	-0.03^***^ (-4.19)	-18.09^***^ (-3.16)	-18.32^***^ (-3.19)
Inactivity	-2.77^***^ (-9.54)	-2.70^***^ (-9.27)	-0.09^***^ (-9.53)	-0.08^***^ (-9.28)	-14.32^**^ (-2.17)	-14.84^**^ (-2.41)
Alcohol	-1.48^***^ (-6.47)	-1.27^***^ (-5.62)	-0.06^***^ (-7.91)	-0.05^***^ (-7.15)	4.40 (0.79)	2.48 (0.42)
Sexual transmitted infections	-0.0002^***^ (-4.98)	-0.0002^***^ (-4.92)	-0.0002^***^ (-4.98)	-0.00004^***^ (-3.83)	0.0003 (0.24)	-0.00002 (-0.03)
Density	-0.00002^**^ (-2.31)	-0.00002^**^ (-2.09)	-0.00004^***^ (-2.76)	-0.00007^**^ (-2.55)	-0.0003 (-1.16)	-0.0003 (-1.18)
Associations	-0.0002 (-0.17)	0.0006 (0.49)	-0.00004 (-1.00)	-0.00002 (-0.41)	-0.03 (-0.60)	-0.03 (-0.63)
Work from home	0.008^***^ (2.57)	0.009^***^ (3.12)	0.0003^***^ (2.60)	0.0003^***^ (3.08)	1.16^***^ (11.41)	1.16^***^ (11.58)
Driving	0.94^***^ (7.47)	1.03^***^ (8.29)	0.03^***^ (6.45)	0.03^***^ (7.22)	16.61^***^ (4.84)	16.93^***^ (4.82)
Broadband	0.40^***^ (4.33)	0.42^***^ (4.45)	0.01^***^ (3.67)	0.01^***^ (3.81)	13.46^***^ (5.59)	13.99^***^ (4.53)
Severe housing problems	-0.46^*^ (-1.90)	-0.30 (-1.21)	-0.01^**^ (-1.97)	-0.01 (-1.34)	-20.86^***^ (-2.64)	-23.45^***^ (-4.16)
Food	4.40^***^ (13.22)	4.66^***^ (14.09)	0.16^***^ (15.85)	0.17^***^ (16.63)	66.98^***^ (6.71)	63.81^***^ (4.74)
Pollution	0.02^***^ (6.70)	0.02^***^ (6.62)	0.0006^***^ (5.09)	0.0006^***^ (5.01)	-0.72^***^ (-6.19)	-0.71^***^ (-6.38)
Number of observations	2736	2733	2736	2733	2307	2305
R^2^	0.9972	0.9972	0.9973	0.9973	0.9057	0.9060

^*^Significant at 10% ^**^Significant at 5% ^***^Significant at 1%

**Table 3:** Results of the estimations (gender inequality)

	Poor mental health days		Frequent mental distress		Suicide	
	GPG	Female poverty	GPG	Female poverty	GPG	Female poverty
Mean relative income	0.21^***^ (5.97)	0.20^***^ (5.70)	0.003^***^ (3.16)	0.003^***^ (3.05)	-2.84^***^ (-3.50)	-2.55^***^ (-3.08)
Inequality	0.07 (1.11)	0.43^***^ (4.02)	-0.0001 (-0.07)	0.01^***^ (3.50)	-4.01 (-1.39)	7.32^**^ (2.18)
Mental health provider	-0.0003 (-1.48)	-0.0002 (-1.11)	-0.00005 (-0.82)	-0.00004 (-0.55)	-0.006 (-0.48)	-0.005 (-0.40)
Uninsured	-0.99^***^ (-5.80)	-0.98^***^ (-5.78)	-0.03^***^ (-6.22)	-0.03^***^ (-6.22)	24.01^***^ (5.14)	23.96^***^ (5.21)
University	-0.20 (-1.31)	-0.17 (-1.10)	-0.002 (-0.49)	-0.003 (-0.57)	-8.80^*^ (-1.93)	-11.27^***^ (-2.61)
Unemployment	0.19 (0.52)	-0.17 (-1.11)	-0.006 (-0.46)	-0.005 (-0.44)	35.09^*^ (1.67)	36.98^**^ (1.69)
Bad health	4.06^***^ (10.69)	4.07^***^ (10.75)	0.15^***^ (12.05)	0.15^***^ (12.05)	-11.59 (-1.06)	-14.73 (-1.25)
Sleep	5.29^***^ (19.26)	5.24^***^ (19.17)	0.14^***^ (16.35)	0.14^***^ (16.25)	18.92^*^ (1.77)	20.67^*^ (1.87)
Smoke	8.32^***^ (24.27)	8.38^***^ (24.65)	0.33^***^ (29.29)	0.33^***^ (29.67)	53.17^***^ (2.94)	51.94^***^ (3.04)
Obesity	-1.52^***^ (-6.17)	-1.59^***^ (-6.50)	-0.03^***^ (-4.19)	-0.03^***^ (-4.44)	-18.09^***^ (-3.16)	-18.90^***^ (-3.32)
Inactivity	-2.69^***^ (-9.27)	-2.76^***^ (-9.53)	-0.08^***^ (-9.28)	-0.08^***^ (-9.51)	-14.58^**^ (-2.26)	-15.49^**^ (-2.37)
Alcohol	-1.28^***^ (-5.56)	-1.32^***^ (-5.87)	-0.05^***^ (-6.98)	-0.06^***^ (-7.41)	5.65 (1.00)	2.94 (0.52)
Sexual transmitted infections	-0.0002^***^ (-4.83)	-0.0002^***^ (-4.78)	-0.0002^***^ (-3.71)	-0.00004^***^ (-3.70)	0.0004 (0.34)	0.0003 (0.28)
Density	-0.00002^**^ (-2.10)	-0.00002^**^ (-2.26)	-0.00007^**^ (-2.50)	-0.00007^***^ (-2.70)	-0.0003 (-1.10)	-0.0003 (-1.32)
Associations	0.0005 (0.44)	0.0001 (0.10)	-0.00002 (-0.43)	-0.00003 (-0.74)	-0.03 (-0.57)	-0.04 (-0.74)
Work from home	0.009^***^ (2.95)	0.008^***^ (2.71)	0.0003^***^ (3.10)	0.0003^***^ (2.76)	1.18^***^ (11.28)	1.14^***^ (11.35)
Driving	1.01^***^ (7.99)	0.93^***^ (7.44)	0.03^***^ (7.10)	0.03^***^ (6.47)	17.42^***^ (5.23)	14.63^***^ (4.11)
Broadband	0.40^***^ (4.22)	0.34^***^ (3.57)	0.01^***^ (3.75)	0.009^***^ (3.03)	14.48^***^ (5.50)	12.26^***^ (5.06)
Severe housing problems	-0.29 (-1.22)	-0.29 (-1.23)	-0.009 (-1.19)	-0.01 (-1.32)	-19.06^**^ (-2.32)	-21.43^***^ (-2.91)
Food	4.71^***^ (14.53)	4.67^***^ (14.51)	0.17^***^ (17.05)	0.17^***^ (17.10)	65.96^***^ (6.17)	66.45^***^ (6.36)
Pollution	0.02^***^ (6.61)	0.02^***^ (6.64)	0.0006^***^ (4.97)	0.0006^***^ (5.03)	-0.72^***^ (-6.18)	-0.72^***^ (-6.20)
Number of observations	2735	2736	2735	2736	2307	2307
R^2^	0.9972	0.9972	0.9973	0.9973	0.9059	0.9059

^*^Significant at 10% ^**^Significant at 5% ^***^Significant at 1%

**Table 4:** Results of the estimations (racial inequality)

	Poor mental health days		Frequent mental distress		Suicide	
	Black poverty	Segregation	Black poverty	Segregation	Black poverty	Segregation
Mean relative income	0.20^***^ (5.38)	0.17^***^ (4.36)	0.003^***^ (2.88)	0.002 (1.56)	-1.97^***^ (-2.67)	-1.68^**^ (-2.23)
Inequality	0.15^***^ (3.18)	0.001^***^ (3.21)	0.004^***^ (2.98)	0.00003^**^ (2.52)	-10.32^***^ (-9.88)	0.03^***^ (2.87)
Mental health provider	-0.0002 (-1.15)	-0.0001 (-0.94)	-0.00003 (-0.59)	-0.00002 (-0.42)	-0.007 (-0.65)	-0.001 (-0.15)
Uninsured	-1.12^***^ (-6.28)	-1.33^***^ (-6.93)	-0.04^***^ (-6.59)	-0.04^***^ (-7.36)	21.59^***^ (5.62)	18.68^***^ (4.66)
University	-0.21 (-1.26)	0.11 (0.62)	-0.002 (-0.47)	0.009^*^ (1.69)	-5.44 (-1.40)	-9.82^**^ (-2.38)
Unemployment	0.21 (0.58)	0.43 (1.14)	-0.003 (-0.29)	0.0002 (0.02)	30.63^***^ (3.13)	1.55 (0.18)
Bad health	3.77^***^ (9.36)	4.02^***^ (9.16)	0.14^***^ (10.79)	0.15^***^ (11.17)	-15.77^*^ (-1.74)	-6.17 (-0.67)
Sleep	4.70^***^ (16.66)	4.21^***^ (13.86)	0.12^***^ (14.15)	0.10^***^ (11.40)	0.47 (0.08)	-4.54 (-0.82)
Smoke	8.67^***^ (23.57)	8.52^***^ (21.13)	0.35^***^ (28.81)	0.35^***^ (26.23)	25.62^***^ (2.80)	30.29^***^ (3.66)
Obesity	-1.66^***^ (-6.31)	-2.15^***^ (-7.69)	-0.04^***^ (-4.51)	-0.05^***^ (-6.14)	-20.09^***^ (-3.83)	-21.11^***^ (-3.99)
Inactivity	-2.05^***^ (-6.58)	-1.18^***^ (-3.51)	-0.06^***^ (-6.77)	-0.04^***^ (-4.26)	-11.13^**^ (-2.08)	-14.41^**^ (-2.49)
Alcohol	-1.15^***^ (-4.78)	-1.21^***^ (-4.65)	-0.05^***^ (-6.08)	-0.05^***^ (-5.21)	-0.12 (-0.02)	-4.76 (-0.95)
Sexual transmitted infections	-0.0002^***^ (-4.89)	-0.0001^***^ (-3.29)	-0.00005^***^ (-3.70)	-0.00002 (-1.50)	0.003^***^ (2.69)	-0.002^**^ (-2.30)
Density	-0.00002^*^ (-1.78)	-0.00001 (-1.60)	-0.00006^**^ (-2.31)	-0.00006^**^ (-2.26)	-0.00009 (-0.49)	-0.0002 (-1.15)
Associations	0.0007 (0.51)	-0.001 (-0.68)	-0.00003 (-0.60)	-0.0001^**^ (-2.42)	0.05 (1.21)	-0.11^**^ (-2.42)
Work from home	0.01^***^ (3.92)	0.009^**^ (2.48)	0.0004^***^ (4.13)	0.0004^***^ (2.98)	0.91^***^ (10.75)	0.68^***^ (6.88)
Driving	1.03^***^ (7.74)	1.29^***^ (9.21)	0.03^***^ (6.62)	0.03^***^ (7.89)	23.21^***^ (7.51)	17.78^***^ (5.25)
Broadband	0.50^***^ (4.58)	0.34^***^ (3.02)	0.01^***^ (3.86)	0.01^***^ (2.74)	2.39 (1.04)	9.49^***^ (3.99)
Severe housing problems	-0.09 (-0.37)	-0.18 (-0.63)	-0.004 (-0.54)	-0.01 (-1.07)	-21.31^***^ (-4.22)	-13.90^**^ (-2.47)
Food	4.70 (13.25)	4.62^***^ (12.64)	0.17^***^ (15.82)	0.17^***^ (15.38)	63.55^***^ (8.02)	55.61^***^ (7.33)
Pollution	0.02 (5.40)	0.02^***^ (3.79)	0.0005^***^ (4.02)	0.0003^***^ (2.59)	-0.49^***^ (-5.50)	-0.38^***^ (-4.06)
Number of observations	2449	1941	2449	1941	2158	1790
R^2^	0.9973	0.9974	0.9974	0.9975	0.9305	0.9338

^*^Significant at 10% ^**^Significant at 5% ^***^Significant at 1%

The first conclusion that can be drawn from the 18 estimates is that the model is robust since there are hardly any significant changes in either the estimated regressors or their significance. Likewise, the quality of the fit is good since the R^2^ ranges between 0.906 and 0.997.

As for the values obtained, in most cases they are those expected a priori. Starting with the variables measuring inequality, the first measure used is the real mean income of the county in relation to that of the state in which it is located. The parameter obtained is highly significant in almost all the estimates made, although the sign changes depending on the measure of mental health. Thus, it is observed that those counties that are richer in relative terms are the ones that suffer more days of poor mental health. However, when these mental health problems become more severe (“frequent mental distress”), the incidence of this variable decreases in value and significance. In the extreme case, i.e., suicide, the sign changes and it is the poorest counties that suffer the most from this problem. This changing result is consistent with what is happening in the economic literature. As Ridley et al. (2020) and Shah et al. (2021) point out, the results obtained in published work on the subject do not allow inferences to be drawn about causality between income and mental health, which hampers opportunities to inform public policy. Thus, for example, while Gresenz et al. (2001) find a strong correlation between individual income level and mental health, although not at the State level, Araya et al. (2003) find no association between income and prevalence of common mental disorders. In any case, the results obtained in our work agree with those obtained by Thomson et al. (2022), who demonstrate the existence of a deterioration in mental health as a result of lower income. It should be taken into account that in richer and therefore more economically dynamic counties, competition is greater and this can lead to some mental distress (Colantone et al., 2019).

In order to study in depth, the effects of inequality on mental health, six measures of inequality have been used for each county, with the aim of analyzing not only inequality in income distribution, commonly used in the literature, but also gender inequality and racial inequality. This allows us to study the effects of inequality, understood in a global way, on the dependent variables, and to test whether greater inequality within the county has effects on mental health. According to the results obtained, the positive (with one exception) and significant sign in 11 of the 18 estimates allows us to conclude that inequality is a determinant of mental health. The greater the inequality within the county, the worse the mental health. In this sense, precarious working conditions, the stress this entails and the comorbidities associated with poverty that may characterize these counties with higher inequality could explain these results (Llosa et al., 2018; Rönnblad et al., 2019). However, analyzing the inequality measures used one by one, these results can be nuanced. The first thing to note is that racial inequality is the most significant (Table 4). In all estimates, the estimated sign is significant. Moreover, in five of the six estimates made, the sign is positive, so that racial inequality leads to worse mental health, as Wallace et al. (2016) also show for the case of the United Kingdom. What is striking is the negative sign obtained when we use suicide as the dependent variable and the percentage of the black population below the poverty line as a measure of inequality. This result invites us to affirm that the black population is less prone to suicide in situations of poverty as Early and Akers (1993), Goldsmith et al. (2002) and more recently Riddell et al. (2018) have already shown. Regarding income inequality (Table 2), although the sign is always positive, it is only significant in 2 of the 6 estimates made. In this sense, the greater the income inequality within the county, the greater the mental problems, although we did not find any significant result for the extreme case of suicide. This inconclusive result is consistent with that reached by Gresenz et al. (2001), who find no relationship between inequality in income distribution and mental health, or that of Yu (2018) who does find a relationship between income inequality and mental health for men, but not for women. Something similar occurs with gender inequality since the estimated regressor is only significant in three of the six estimates made, although in this case we can indeed state that gender inequality harms mental health and is even a determinant of suicide. These results confirm the findings of McAllister et al. (2018), according to which better mental health is related to lower gender inequality. Mar et al. (2022) also find strong evidence for the relationship between gender inequality, mental health, and suicide.

There are other variants of inequality that can also affect mental health. One of them is health. In this regard, two variables have been used. First, we have employed access to mental health care measured through mental health providers. It is important to note that about 30% of the population lives in a county designated as a mental health professional shortage area (HRSA, 2022). However, the results we obtain are not significant, so we cannot draw conclusions about whether more mental health care facilities lead to a reduction in potential mental disorders. Therefore, we use a second variable, “uninsured”, to analyze whether health coverage, or lack thereof, influences mental health. The results obtained are significant, although different for the extreme case of suicide. Thus, in the case of poor mental health or frequent mental disorders, the parameter obtained is negative, which implies that the higher the percentage of the population that is not insured, the fewer the mental health problems. This result, a priori surprising, can be explained by the high health costs in the USA, which can lead the population without health coverage not to see a specialist when suffering from some type of disorder (Carter et al., 2020). However, when the disease worsens and leads individuals to the extreme solution of suicide, the sign changes and becomes positive, with the uninsured population suffering more from the most severe mental problems, as also shown by Johnson and Brookover (2020), and Ong et al. (2021).

Another form of inequality that can affect mental health is related to education. In this work we have used the percentage of the population with university studies since in the USA there is great inequality in access to higher education (Jerrim et al., 2015). The results obtained show a negative sign, although only significant in 7 of the 18 estimates made. This would show that higher education promotes better mental health (Jiang et al., 2020), since there is a certain correlation between the level of education attained and a better job, better life habits and a better health status. It is worth noting that when we use suicide as a dependent variable, education is significant in five of the six estimates made, making education a key factor in the fight against very serious mental disorders (Lorant et al., 2018).

As mentioned above, more education implies a higher probability of finding a job and thus covering all those material needs whose lack can lead to a deterioration of mental health. This is why we use the “unemployment” variable, which also reflects the inequalities between counties in terms of labor markets. However, the parameter obtained is only significant when suicide is used as the dependent variable. The positive sign allows us to conclude that a higher unemployment rate implies a higher suicide rate, as also shown by Amiri (2021).

From here, the next group of variables that have been studied refer to the health status and life habits. First, we analyzed whether there was any relationship between physical and mental health status. The results obtained, significant in all cases except for suicide, show that there is a direct relationship between both variables, i.e., poor physical health leads to poor mental health (Ohrnberger et al., 2017; Luo et al., 2020). However, as we have discussed above, when we use suicide as the dependent variable, the parameter ceases to be significant, contrary to what most of the economic literature says (Fairweather et al., 2006; Phillips & Hempstead, 2022; Qin et al., 2022). Even so, as Fiske et al. (2008) argue, the relationship between poor health and suicide tends to occur in older population groups. Furthermore, according to Ahmedani et al. (2017) it is important to nuance which determinants of physical health status most affect mental health and suicide. These authors find that lack of sleep is a key factor. This is why we included the variable “sleep” in our analysis. The results obtained show a positive and significant relationship between the percentage of people with sleep problems and the three variables used to measure mental health problems. Therefore, we can affirm that insomnia is a risk factor for mental health as also shown by Chattu et al. (2018), Sullivan and Ordiah (2018), and Merikanto and Partonen (2021) among others.

Other variables that reflect the lifestyle of the population have been included in this analysis. Thus, the estimated parameter for the variable “smoke” is always positive and highly significant, whereby the higher the percentage of smokers the worse the mental health, as also argued by Ferreira et al. (2019). There is a common perception that smoking generally helps people to manage stress and can be a form of “self-medication” in people with mental health problems, although this addiction, like others, generates withdrawal symptoms that worsen mental health (Taylor et al., 2021). However, when we estimate the relationship between excessive alcohol consumption and mental health, the result obtained is surprising. The sign is negative and significant in all estimates except when we use suicide as the dependent variable. In this case, the estimated parameter is not significant. Therefore, we cannot affirm that excessive alcohol consumption is a risk factor for mental health. In this regard, Li et al. (2022) also conclude that, for certain population groups, alcohol consumption is a protective factor against mental disorders. On the other hand, the economic literature has also analyzed the relationship between obesity and physical activity on mental health (Avila et al., 2015). The results obtained in our work show a negative and significant sign for these two variables. Therefore, we cannot affirm that those counties with a higher percentage of obese people and those who report not doing any physical activity have greater mental problems. In this sense, Biddle et al. (2019) also do not obtain evidence of a causal association between physical activity and mental health. To our knowledge, obesity and lack of physical activity would not be a cause but a consequence of mental disorders, as Van der Valk et al. (2018) also demonstrate. In fact, Rajan and Menon (2017) point to a bidirectional relationship between obesity and mental health. Finally, the variable “STI” was used as a proxy for sexual activity. The results obtained, significant in 12 of the 18 estimates made, show that sexual activity reduces mental disorders (Brody, 2010; Mollaioli et al., 2021; Gianotten, 2021).

The economic literature has discussed in depth the relationship between social isolation and mental disorders (Wang et al., 2017). This paper aims to delve into the relationship between the two concepts with the use of five variables. Thus, first, we employ the variable measuring population density to test whether denser counties have lower mental problems. Epidemiological studies show that the risk of serious mental illness is higher in cities than in rural areas, where population density is lower (Gruebner et al., 2017) since higher density is associated with lower social contacts (Giacco et al., 2022). However, the negative and significant sign in 11 of the 18 estimates made allow us to affirm that in those counties with higher population per km^2^ makes personal relationships closer and fosters better mental health. This result, contrary to that shown by other authors, perhaps requires a more specific analysis of this relationship, as Lai et al. (2021) have done. These authors conclude that rather than population density per se, it is urban design that determines the relationship between density and mental health. In fact, the evidence of the impacts of increasing urban densification on loneliness and social isolation in humans is still inconclusive. For this reason, we use other variables that reflect social isolation. Thus, for the case of the variable “associations”, we did not find a significant relationship with respect to mental health, making it unclear whether these types of social associations improve mental health (Wakefield et al., 2019). Regarding teleworking, a variable that reflects the lack of social contact in the workplace, the positive and highly significant sign obtained in all the estimates made shows that, indeed, a higher percentage of teleworkers and, therefore, greater social isolation, leads to greater mental disorders and even the extreme case of suicide. Authors such as Mann and Holdsworth (2003) and De Sio et al. (2021) have already demonstrated the harmful effects of teleworking on mental health. This result is confirmed when we use the variable “driving” which reflects the percentage of people who drive alone every day to work. Again, this variable is used as a proxy for social isolation, and the results obtained (positive and highly significant sign in all the estimates made) allow us to conclude that social isolation is a key risk factor for mental disorders. Finally, Internet access was used as a proxy variable for Internet use at home and, therefore, less social contact. The positive and highly significant sign shows that the higher the use of the Internet and social networks, the higher the probability of suffering from mental disorders. These results agree with those obtained by Grant et al. (2019), Arzani-Birgani et al. (2021), and Golin (2022).

Other problems that can cause mental distress are material problems in a household and food insecurity. Now, which of the two generates more distress? Our analysis reveals that food insecurity is a risk factor for mental health, whereas we did not find a clear result for the variable reflecting severe household problems. Probably, as suggested by Singh et al. (2019), it would be necessary to analyze each of the household problems to see how they individually impact mental health. The harmful effects of food insecurity on mental health have also been shown by authors such as Pourmotabbed et al. (2020) among others.

Finally, the effect of pollution on mental health has been analyzed. The results obtained allow us to affirm that pollution is a risk factor for mental health, although in the extreme case of suicide, the sign changes, so we cannot affirm that those more polluting counties suffer from a higher suicide rate (Heo et al., 2021). As Ventriglio et al. (2021) argue, the impact of pollution on public health is well known, but the association between environmental pollutants and mental health has been little analyzed, and most of these yield inconclusive results. In any case, our results confirm the theses of Chen et al. (2018) and Yang et al. (2021).

## Conclusion

Is inequality a risk factor for mental health? What lifestyle habits worsen mental health the most? What are the effects of the implementation of teleworking on mental disorders? To these and other questions we have tried to answer in this paper. Using a sample of 2,735 U.S. counties, a cross-sectional linear model has been estimated. The results obtained allow us to conclude that income is a key factor determining mental health status. While wealthier counties are more likely to suffer from mild mental disorders, when these worsen to the extreme case of suicide, it is the poorer counties that suffer the most. However, it is not severe housing problems that lead to the extreme deterioration of mental health, but rather the food insecurity suffered by poor families. For this reason, public assistance programs to meet the most basic needs are necessary in this country, since there are many households that suffer as a result of the strong inequality that exists. In fact, inequality, understood in a broad sense, is a key determinant of mental health. For this reason, public policies must be implemented to mitigate income differences, and to fight against gender inequality and all types of racial discrimination. In addition, the health coverage network should continue to be extended to the entire American population, and a scholarship plan should be promoted to allow greater access to higher education. All of this will result in fewer mental health problems.

On the other hand, because of the COVID-19 pandemic, many companies have adapted to increased teleworking. This, although it has served to prevent the virus, is a problem for mental health, as our results show. Thus, we show that the social isolation produced by teleworking, driving alone to work and the increased use of the Internet at home are seriously damaging to mental health. Therefore, a more detailed analysis of the pros and cons of promoting teleworking should be carried out by policy makers and companies.

The third pillar on which we have based our analysis of mental disorders is healthy lifestyle habits. In this regard, we emphasize that lack of sleep and addiction to tobacco are risk factors for mental health, while sexual activity is a good medicine against this type of disorder.

Finally, pollution also harms mental health. Thus, we find yet another argument for policymakers to step up measures to combat climate change.

However, this work is not without limitations. The first limitation is the lack of post-COVID-19 mental health data, which would have allowed us to draw stronger conclusions about the incidence of social isolation and mental health. The data we have been able to work with refer to 2019, so future updates of the data will allow the effect of COVID-19 to be included and will undoubtedly improve the analysis performed. Likewise, it would have been desirable to have more measures of both gender and racial inequality to make the analysis more robust. Thus, for example, it would have been very interesting to have available measures of gender inequality by US counties such as the Global Gender Gap, Gender Inequality Index or the Social Watch Gender Equity Index. Likewise, having a database on multidimensional racial inequality, as calculated by Rohde and Guest (2013) might have solved some of the problems of non-significance that we have encountered.

## Data Availability

All the data used in this work are available in the web pages listed in Table 1.
